# Rhizosphere Growth-Promoting Bacteria Enhance Oat Growth by Improving Microbial Stability and Soil Organic Matter in the Saline Soil of the Qaidam Basin

**DOI:** 10.3390/plants14131926

**Published:** 2025-06-23

**Authors:** Xin Jin, Xinyue Liu, Jie Wang, Jianping Chang, Caixia Li, Guangxin Lu

**Affiliations:** College of Agriculture and Animal Husbandry, Qinghai University, Xining 810016, China; 18894310895@163.com (X.J.); 17856838703@163.com (X.L.); wangjie422022@163.com (J.W.); c223663@126.com (J.C.); lcxia1314@126.com (C.L.)

**Keywords:** alpine forage, PGPR, comprehensive growth index, microbial community stability

## Abstract

The Qinghai–Tibet Plateau, a critical ecological barrier and major livestock region, faces deteriorating grasslands and rising forage demand under its harsh alpine climate. Oat (*Avena sativa* L.), valued for its cold tolerance, rapid biomass accumulation, and ability to thrive in nutrient-poor soils, can expand winter feed reserves and partly alleviate grazing pressure on native rangelands. However, genetic improvement alone has not been sufficient to address the environmental challenges. This issue is particularly severe in the Qaidam Basin, where soil salinization, characterized by high pH, poor soil structure, and low nutrient availability, significantly limits crop performance. Rhizosphere growth-promoting bacteria (PGPR) are environmentally friendly biofertilizers known to enhance crop growth, yield, and soil quality, but their application in the saline soil of the Qaidam Basin remains limited. We evaluated two PGPR application rates (B1 = 75 kg hm^−2^ and B2 = 150 kg hm^−2^) on ‘Qingtian No. 1’ oat, assessing plant growth, soil physicochemical properties, and rhizosphere microbial communities. The results indicated that both treatments significantly increased oat productivity, raised the comprehensive growth index, augmented soil organic matter, and lowered soil pH; B1 chiefly enhanced above-ground biomass and fungal community stability, whereas B2 more strongly promoted root development and bacterial community stability. Structural equation modeling showed that PGPR exerted direct effects on the comprehensive growth index and indirect effects through soil and microbial pathways, with soil properties contributing slightly more than microbial factors. Notably, rhizosphere organic matter, fungal β-diversity, and overall microbial community stability emerged as positive key drivers of the comprehensive growth index. These findings provide a theoretical basis for optimizing PGPR dosage in alpine forage systems and support the sustainable deployment of microbial fertilizers under saline soil conditions in the Qaidam Basin.

## 1. Introduction

The Qinghai–Tibet Plateau, a globally significant ecological barrier and pastoral region, faces dual challenges: persistent grassland degradation and a rapidly increasing demand for livestock production [1,2]. Harsh alpine climatic conditions limit the productivity of traditional forage species while overgrazing exacerbates the fragility of grassland ecosystems [3]. Oat (*Avena sativa* L.), due to its strong cold tolerance, rapid biomass accumulation, and adaptability to nutrient-deficient soils [4], has emerged as a widely promoted crop in the region. Its cultivation not only enhances the availability of winter supplemental forage but also partially alleviates grazing pressure on natural grasslands [5]. Although genetic improvement has contributed significantly to enhancing crop adaptability and productivity, it alone remains insufficient to fully overcome the severe environmental constraints [6]. The challenges associated with soil salinity are particularly severe in the Qaidam Basin, where soils are characterized by high pH, poor physical structure, and low nutrient availability [7]. These adverse soil conditions significantly hinder crop growth and yield performance [8], highlighting the urgent need to enhance forage production potential through the combination of superior cultivars and integrated agronomic practices. Such efforts are essential to improve forage quality and productivity and to support the sustainable development of both agriculture and animal husbandry in the Qaidam Basin.

Microbial inoculants, as environmentally friendly alternatives to chemical fertilizers, have demonstrated substantial potential in enhancing crop growth, yield, and soil quality in recent years [9,10]. These biofertilizers act through biological nitrogen fixation, solubilization of phosphate and potassium, phytohormone production (e.g., auxins, gibberellins, cytokinins), and the induction of systemic resistance. These processes enhance plant tolerance to abiotic stresses and ultimately improve plant development and yield performance [11]. Studies have shown that microbial inoculants significantly enhance aboveground biomass, improve forage quality, and stimulate root system development while also improving the rhizosphere environment [12,13,14]. Moreover, microbial inoculants improve soil physicochemical properties by increasing organic matter content, enhancing soil aggregation, and increasing the bioavailability of key nutrients (e.g., ammonium nitrogen, available phosphorus), thereby improving soil aeration and water-holding capacity [15]. More importantly, microbial inoculants can reshape soil microbial community composition, enhance microbial diversity and metabolic activity, and improve the functional stability and resilience of the soil micro-ecosystem [16,17]. Accumulating evidence indicates that plant–microbe–soil interactions play a pivotal role in regulating plant adaptation to harsh environments [18,19]. However, systematic investigations into how microbial inoculants influence forage growth under saline–alkaline stress remain limited [20,21]. In particular, little is known about how microbial inoculants affect plant performance, soil physicochemical properties, and microbial community composition under the saline–alkaline stress typical of the Qaidam Basin. Therefore, elucidating the role of microbial inoculants in improving forage productivity under the saline soil conditions of the Qaidam Basin is critical for establishing a theoretical foundation for their effective application in this severely stressed environment.

In this study, the cold-tolerant oat variety ‘Qingtian No. 1’, cultivated in the saline soil of the Qaidam Basin, Qinghai Province, was used as the experimental crop. Two application rates of plant growth-promoting rhizobacteria (PGPR), 75 kg·hm^−2^ and 150 kg·hm^−2^, were applied to systematically address the following research questions: (1) whether PGPR application promotes oat growth and whether the effects are consistent across different application rates; (2) the effects of PGPR on the physicochemical properties of rhizosphere soil; (3) the effects of PGPR on the diversity and stability of rhizosphere microbial communities; and (4) how PGPR-induced changes in rhizosphere soil and microbial communities regulate oat growth. By addressing these questions, this study aims to establish a theoretical foundation for the application of PGPR to enhance oat production under saline soil conditions in the Qaidam Basin.

## 2. Results

### 2.1. Oat Growth Performance and Root Development

**Figure 1:**
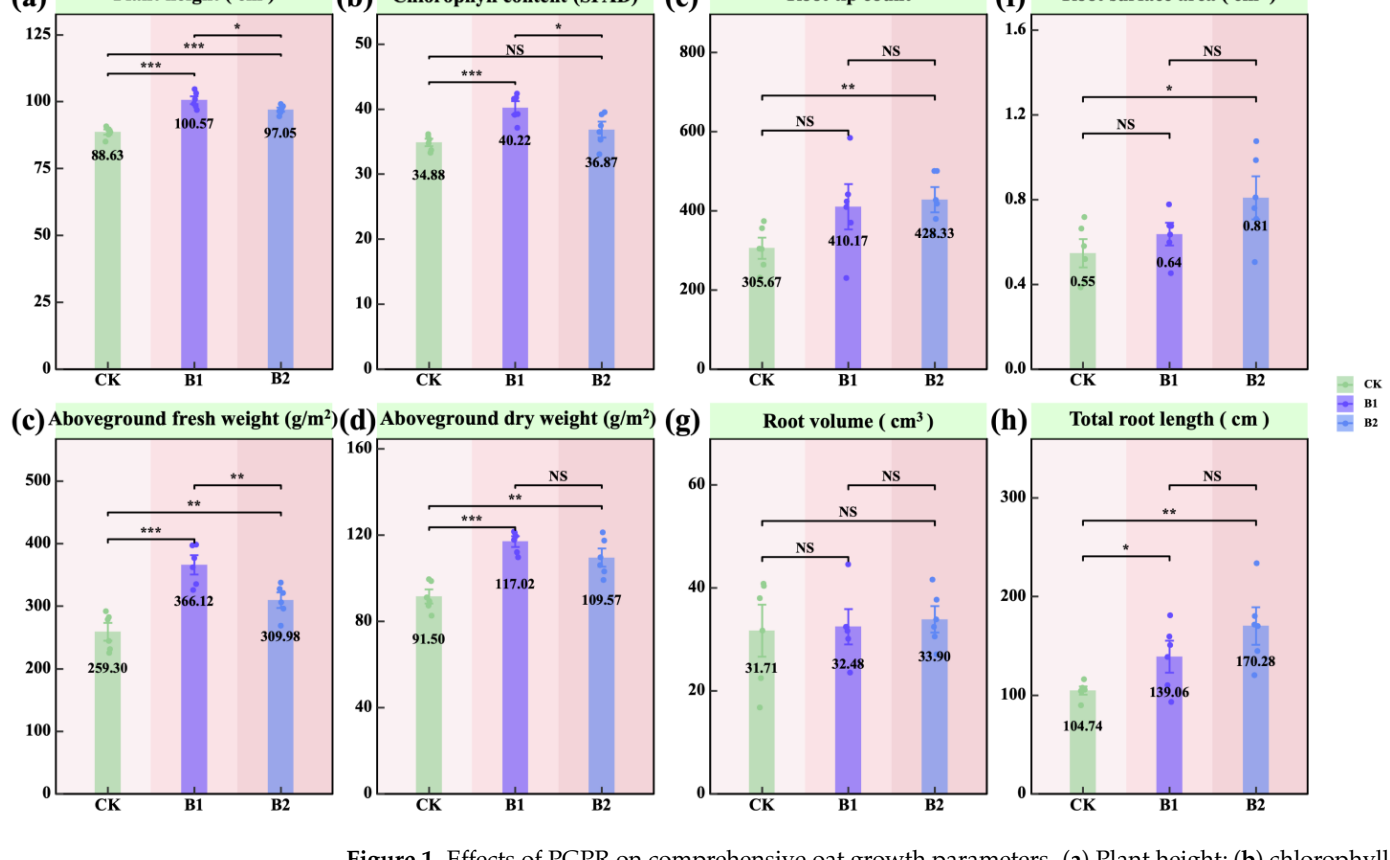
Effects of PGPR on comprehensive oat growth parameters. (**a**) Plant height; (**b**) chlorophyll content; (**c**) aboveground fresh weight; (**d**) aboveground dry weight; (**e**) root tip count; (**f**) root surface area; (**g**) root volume; (**h**) total root length. The values in panels (**a**–**h**) represent means. Pairwise comparisons among treatments—CK (*n* = 6), B1 (*n* = 6), and B2 (*n* = 6)—were conducted using independent two-sample *t*-tests (df = 10), and final *p*-values were adjusted using the Bonferroni correction. Note: the green background in the title indicates plants. Asterisks denote statistical significance (^NS^ *p* > 0.05; * *p* < 0.05; ** *p* < 0.01; *** *p* < 0.001).

Plant height, aboveground fresh weight, and aboveground dry weight were significantly higher in both B1 and B2 treatments compared to CK (*p* < 0.01; Figure 1a,c,d). Additionally, the SPAD value in the B1 treatment was significantly higher than in both CK and B2 (*p* < 0.05; Figure 1b). Increased application rates of PGPR enhanced root development. Specifically, root tip number, root surface area, and total root length were significantly greater in B2 compared to CK (*p* < 0.05; Figure 1e,f,h). These results suggest that B1 was more effective in promoting aboveground oat growth, whereas B2 had a stronger effect on root system development.

### 2.2. Rhizosphere Soil Physicochemical Properties

**Figure 2:**
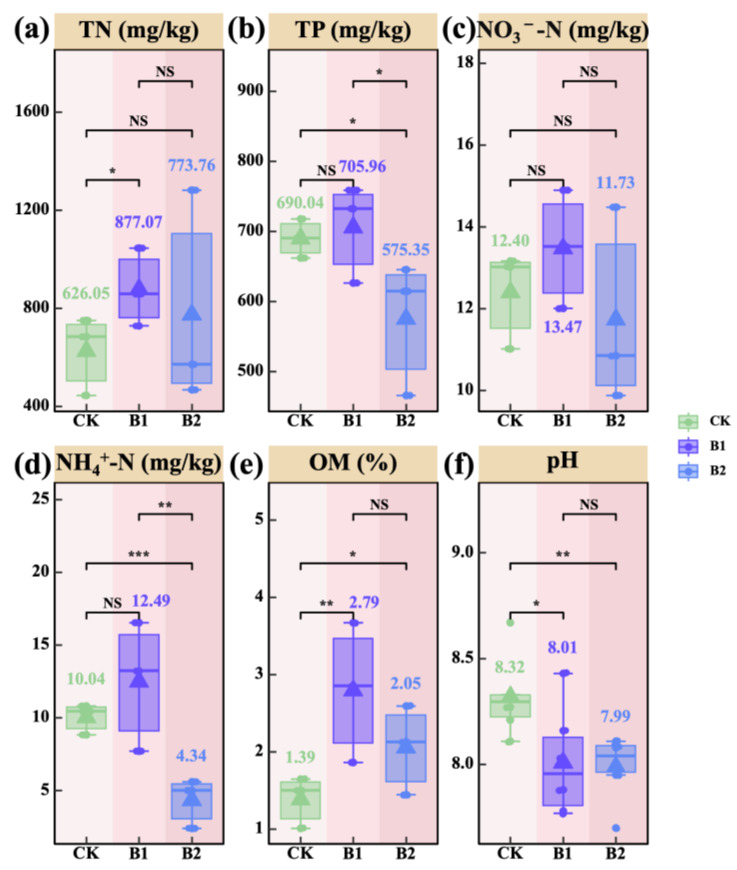
Effects of PGPR on the physicochemical properties of rhizosphere soil. (**a**) Soil total nitrogen (TN); (**b**) soil total phosphorus (TP); (**c**) soil nitrate nitrogen (NO_3_^−^-N); (**d**) soil ammonium nitrogen (NH_4_^+^-N); (**e**) soil organic matter (OM); (**f**) soil pH (pH). The values in figures (**a**–**f**) indicate means. Pairwise comparisons among treatments—CK (*n* = 6), B1 (*n* = 6), and B2 (*n* = 6)—were conducted using independent two-sample *t*-tests (df = 10), and final *p*-values were adjusted using the Bonferroni correction. Note: the brown background in the title indicates rhizosphere soil. Asterisks indicate statistical significance (^NS^ *p* > 0.05; * *p* < 0.05; ** *p* < 0.01; *** *p* < 0.001).

Total nitrogen (TN) and organic matter (OM) contents were significantly higher in the B1 treatment compared to CK (*p* < 0.05; Figure 2a,e). In contrast, total phosphorus (TP) and ammonium nitrogen (NH_4_^+^-N) levels were significantly lower in the B2 treatment compared to CK (*p* < 0.05; Figure 2b,d). Higher application rates of PGPR significantly reduced soil pH (*p* < 0.05; Figure 2f). These findings suggest that B1 promotes the accumulation of soil TN and OM, whereas B2 suppresses the accumulation of TP and NH_4_^+^-N. Moreover, increased PGPR application significantly lowered soil pH, thereby alleviating rhizosphere saline soil environment.

### 2.3. Diversity, Structure, and Stability of Rhizosphere Microbial Communities

**Figure 3:**
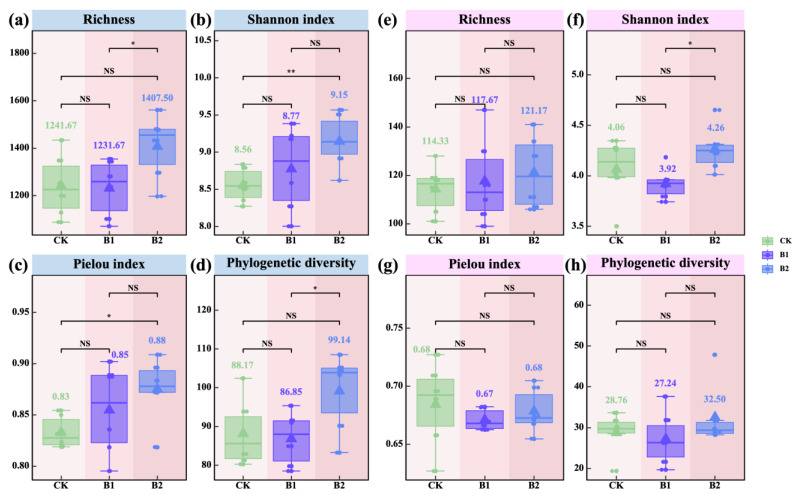
Effects of PGPR on the α-diversity of rhizosphere microbial communities. (**a**–**d**) represent rhizosphere soil bacterial communities, and (**e**–**h**) represent rhizosphere soil fungal communities. The values in figures (**a**–**h**) indicate means. Pairwise comparisons among treatments—CK (*n* = 6), B1 (*n* = 6), and B2 (*n* = 6)—were conducted using independent two-sample *t*-tests (df = 10), and final *p*-values were adjusted using the Bonferroni correction. Note: the blue background in the title indicates rhizosphere soil bacteria; the pink background indicates rhizosphere soil fungi. Asterisks indicate statistical significance (^NS^ *p* > 0.05; * *p* < 0.05; ** *p* < 0.01).

As the application rate of PGPR increased, the richness and phylogenetic diversity of rhizosphere bacterial communities initially declined and then increased, with B2 showing significantly higher values than B1 (*p* < 0.05; Figure 3a,d). In contrast, the Shannon index and Pielou index of bacterial communities gradually increased, with B2 significantly higher than CK (*p* < 0.05; Figure 3b,c). Similarly, with increasing PGPR application rates, the Shannon index, Pielou index, and phylogenetic diversity of rhizosphere fungal communities showed a decrease followed by an increase, while the richness index continuously increased. Except for the Shannon index, which was significantly higher under B2 compared to B1, no significant differences were observed among the other treatments (Figure 3e–h). These results suggest that the B2 treatment effectively enhanced the species richness and phylogenetic diversity of the oat rhizosphere bacterial community and improved community evenness. For fungal communities, B2 primarily promoted an increase in overall community diversity.

**Figure 4:**
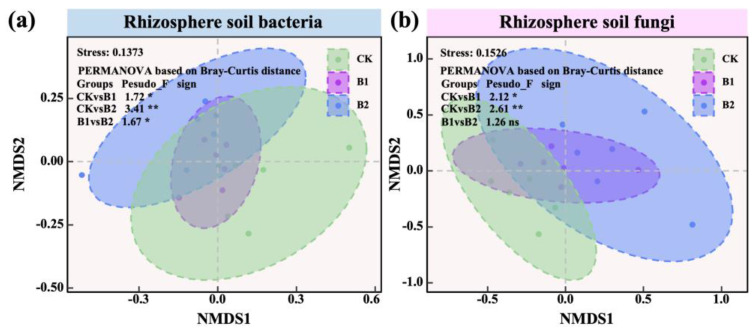
Effects of PGPR on rhizosphere microbial communities (based on non-metric multidimensional scaling; NMDS). (**a**) Rhizosphere soil bacterial community. (**b**) Rhizosphere soil fungal community. Differences in community composition among CK, B1, and B2 were assessed using PERMANOVA (based on Bray–Curtis distance), sample sizes: CK (*n* = 6), B1 (*n* = 6), and B2 (*n* = 6). Note: the blue background in the title indicates rhizosphere soil bacteria; the pink background indicates rhizosphere soil fungi. Asterisks indicate statistical significance (^ns^ *p* > 0.05; * *p* < 0.05; ** *p* < 0.01).

The community structures of rhizosphere bacteria and fungi were assessed via non-metric multidimensional scaling (NMDS). Bacterial communities in both B1 and B2 treatments differed significantly from CK (PERMANOVA: CK vs. B1 = 1.72 *, CK vs. B2 = 3.41 **; Figure 4a), and a significant difference was also observed between B1 and B2 (B1 vs. B2 = 1.67 *; Figure 4a). Similarly, rhizosphere fungal communities in both B1 and B2 treatments were significantly distinct from CK (PERMANOVA: CK vs. B1 = 2.12 *, CK vs. B2 = 2.61 **; Figure 4b). These findings suggest that PGPR application substantially reshaped the rhizosphere microbial community structure, with B2 exerting a stronger influence on community composition.

**Figure 5:**
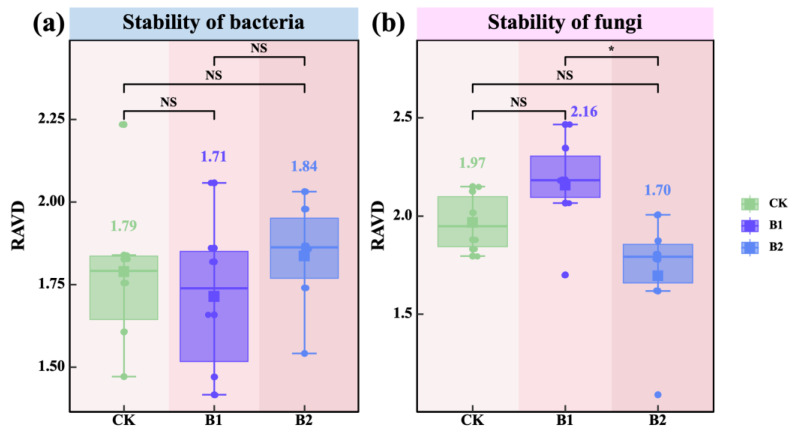
Effects of PGPR on the stability of rhizosphere microbial communities. (**a**) Stability of the rhizosphere soil bacterial community. (**b**) Stability of the rhizosphere soil fungal community. Note: the blue background in the title indicates rhizosphere soil bacteria; the pink background indicates rhizosphere soil fungi. The values in figures (**a**,**b**) indicate means. Pairwise comparisons among treatments—CK (*n* = 6), B1 (*n* = 6), and B2 (*n* = 6)—were conducted using independent two-sample *t*-tests (df = 10), and final *p*-values were adjusted using the Bonferroni correction. Note: the blue background in the title indicates rhizosphere soil bacteria; the pink background indicates rhizosphere soil fungi. Asterisks indicate statistical significance (^NS^ *p* > 0.05; * *p* < 0.05).

The stability of rhizosphere soil microbial communities in response to PGPR application was analyzed. The results showed that with increasing PGPR application rates, the stability (RAVD) of rhizosphere soil bacterial communities consistently increased (Figure 5a). In contrast, fungal community stability initially increased and then declined, with B1 exhibiting significantly higher stability than B2 (*p* < 0.05; Figure 5b). These findings suggest that the B1 treatment was more effective in enhancing fungal community stability, whereas the B2 treatment was more beneficial for improving bacterial community stability.

### 2.4. Comprehensive Oat Growth and Regulatory Pathways of Key Driving Factors

**Figure 6:**
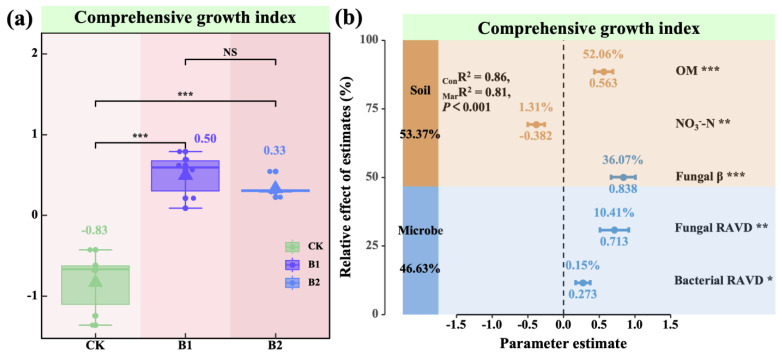
Differences in comprehensive growth index of oat and the relative contributions of key driving factors. (**a**) Variations in oat comprehensive growth index. The values in the figure (**a**) indicate means. Pairwise comparisons among treatments—CK (*n* = 6), B1 (*n* = 6), and B2 (*n* = 6)—were conducted using independent two-sample *t*-tests (df = 10), and final *p*-values were adjusted using the Bonferroni correction. Note: The triangles in the figure represent the means of different treatment groups. (**b**) Relative contributions of habitat key factors to changes in comprehensive growth index. _Mar_R^2^ (marginal R^2^) represents the proportion of variance explained by fixed effects alone, whereas _Con_R^2^ (conditional R^2^) denotes the variance explained by both fixed and random effects. The mean parameter estimates (standardized regression coefficients) of the predictors and their associated 95% confidence intervals, the relative importance of each predictor. Soil organic matter (OM), soil nitrate nitrogen (NO_3_^−^-N), fungal β-diversity (Fungal β), fungal community stability (Fungal RAVD), and bacterial community stability (Bacterial RAVD). Asterisks denote statistical significance (^NS^ *p* > 0.05; * *p* < 0.05; ** *p* < 0.01; *** *p* < 0.001).

The results for the comprehensive plant growth index indicated that with increasing PGPR application rates, the oat comprehensive growth index initially increased and then decreased, with both B1 and B2 treatments being significantly higher than the CK treatment (*p* < 0.001; Figure 6a), and no significant difference was observed between B1 and B2 (*p* > 0.05; Figure 6a). The mixed-effects model showed that soil and microbial factors together explained 86% of the variation in the comprehensive growth index (_Mar_R^2^ = 0.81, *p* < 0.001; Figure 6b), with soil contributing 53.37% and microbial factors contributing 46.63%. Further analysis revealed that bacterial community stability (Bacterial RAVD), fungal community stability (Fungal RAVD), fungal β-diversity (Fungal β), and soil organic matter (OM) significantly promoted the comprehensive oat growth index (*p* < 0.05; Figure 6b), whereas nitrate nitrogen (NO_3_^−^-N) had a significant negative effect (*p* < 0.01; Figure 6b). These results suggest that PGPR application markedly enhanced the comprehensive growth performance of oats by optimizing both soil physicochemical properties and rhizosphere microbial community structure. Among these factors, organic matter content, fungal β-diversity, and fungal community stability were identified as the key drivers influencing oat growth.

**Figure 7:**
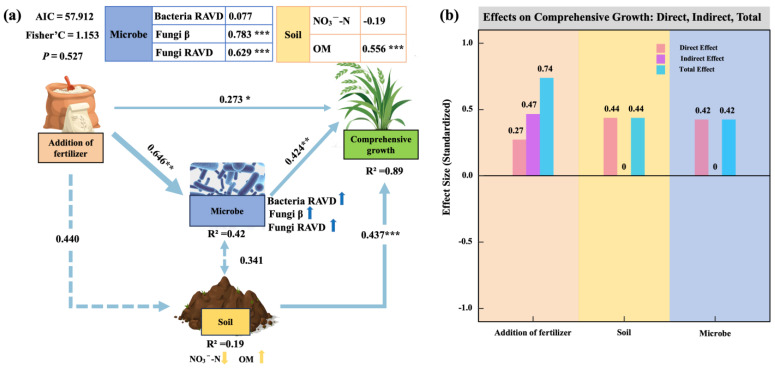
Regulatory pathways influencing comprehensive oat growth performance. (**a**) Structural equation modeling (SEM) reveals the regulatory pathways. (**b**) Quantitative contributions of fertilizer, soil properties, and microbial communities to the comprehensive oat growth index, including their direct, indirect, and total effects. Path coefficients represent standardized effect sizes. Blue lines indicate positive effects, and line thickness reflects effect strength. Solid lines denote statistically significant relationships, while dashed lines indicate non-significant correlations. The arrows following the indicators in the figure indicate positive influence when pointing upwards and negative influence when pointing downwards. Asterisks denote statistical significance (* *p* < 0.05; ** *p* < 0.01; *** *p* < 0.001).

A structural equation model (SEM) was constructed using key predictors identified from the mixed-effects model (Figure 7a), explaining 89% of the variation in the comprehensive oat growth index (R^2^ = 0.89). PGPR application positively influenced rhizosphere microbial communities (β = 0.646, *p* < 0.01; Figure 7a), rhizosphere soil properties (β = 0.440, *p* < 0.01; Figure 7a), and the comprehensive growth index (β = 0.273, *p* < 0.05; Figure 7a). Among the mediating pathways, the effect of rhizosphere soil on oat growth (β = 0.437, *p* < 0.001; Figure 7a) was slightly stronger than that of the microbial community (β = 0.424, *p* < 0.01; Figure 7a). Further analysis showed that fungal community stability (Fungal RAVD), fungal β-diversity (Fungal β), and soil organic matter (OM) had strong positive effects on oat growth (*p* < 0.001; Figure 7a), while bacterial community stability (Bacterial RAVD) also contributed positively. In contrast, nitrate nitrogen (NO_3_^−^-N) was negatively associated with oat growth (Figure 7a). The direct, indirect, and total effects of PGPR application, rhizosphere soil properties, and microbial variables were further quantified (Figure 7b). Total effect sizes ranked as follows: PGPR application (0.74) > soil properties (0.44) > rhizosphere microbiome (0.42) (Figure 7b). These findings indicate that PGPR enhances oat growth both directly and indirectly by modulating rhizosphere soil conditions and microbial community structure, thereby jointly promoting plant development.

## 3. Discussion

### 3.1. Effects of PGPR on Oat Growth and Root Development

We evaluated the effects of plant growth-promoting rhizobacteria (PGPR) on oat aboveground and root growth using two application rates, 75 kg·hm^−2^ (B1) and 150 kg·hm^−2^ (B2), alongside a non-inoculated control (CK). PGPR application positively influenced oat growth at both application rates, although the degree of promotion differed between aboveground and root traits depending on the application rate.

For aboveground growth, both B1 and B2 significantly increased plant height, fresh weight, and dry weight (*p* < 0.01; Figure 1a,c,d), suggesting enhanced plant vigor and biomass accumulation [22,23]. Notably, the SPAD value was significantly higher under B1 than under CK and B2 (*p* < 0.05; Figure 1b), indicating that a lower PGPR application rate was more favorable for enhancing chlorophyll content. This aligns with earlier reports suggesting that rhizosphere bacteria can enhance plant performance partly by modulating chlorophyll metabolism and SPAD index under both full and deficit irrigation conditions [24]. Regarding root development, root tip number, root surface area, and total root length increased significantly with higher PGPR application rates, with the most pronounced effects observed under B2 (*p* < 0.05; Figure 1e,f,h). This finding indicates that higher PGPR application rates more effectively promoted root system expansion, potentially through enhanced root exudate secretion [25], activation of the rhizosphere microenvironment, and stimulation of root cell division [9,26,27].

### 3.2. Effects of PGPR on Rhizosphere Soil Nutrients and pH

In terms of soil nutrients, B1 significantly increased soil total nitrogen (TN) and organic matter (OM) contents compared to CK (*p* < 0.05; Figure 2a,e), suggesting that an appropriate PGPR application rate facilitates nitrogen accumulation and organic matter transformation. This effect may be attributed to the elevated metabolic activity of functional microbes within the inoculants, which enhanced rhizosphere organic matter decomposition and promoted nitrogen cycling and the accumulation of stable organic compounds [28,29]. In contrast, B2 significantly reduced soil total phosphorus (TP) and ammonium nitrogen (NH_4_^+^-N) levels (*p* < 0.05; Figure 2b,d), indicating that higher PGPR application rates may enhance plant nutrient uptake and promote the transformation of available phosphorus and nitrogen, thereby reducing their accumulation in the soil [30]. Regarding soil pH, a significant decrease was observed with increasing PGPR application rates, with a more pronounced reduction under B2 (*p* < 0.05; Figure 2f). This reduction could be attributed to biological acidification, whereby rhizosphere-promoting bacteria secrete acidic metabolites and dissolve soil calcium carbonate, effectively lowering the pH of highly alkaline soils [31].

### 3.3. Effects of PGPR on the Diversity, Structure, and Stability of Rhizosphere Microbial Communities

This study further explored the effects of different PGPR application rates on the diversity, structure, and stability of oat rhizosphere microbial communities. The results revealed that PGPR application significantly altered the composition and diversity of both bacterial and fungal communities, exhibiting clear differences that depended on the application rate and the type of microbial community.

With increasing PGPR application rates, bacterial richness and phylogenetic diversity (PD) displayed a unimodal pattern (Figure 3a,d), whereas the Shannon index and Pielou index increased continuously (Figure 3b,c). Notably, under the higher application rate (B2), bacterial diversity and evenness were further enhanced (Figure 3b,c), indicating that B2 promoted bacterial diversification in the rhizosphere [32], with earlier reports showing that increased PGPR input can elevate bacterial evenness and diversity through microhabitat modification and resource redistribution [33]. Fungal communities exhibited a slightly different response (Figure 3e–h): Shannon diversity, Pielou index, and PD displayed a unimodal pattern, while species richness increased steadily with higher PGPR application rates. Although B2 did not significantly enhance all diversity metrics, it positively affected richness and specific aspects of fungal diversity, significantly elevating the Shannon index and suggesting a potential for increased fungal community complexity, similar to what was observed in mycorrhiza–PGPR co-inoculated revegetation systems under semi-arid stress [34]. NMDS and PERMANOVA analyses confirmed that PGPR application significantly reshaped the composition of rhizosphere microbial communities. Both B1 and B2 treatments differed significantly from CK, and significant differences were also observed between B1 and B2 (Figure 4a). These findings highlight the strong remodeling capability of PGPR in rhizosphere communities [35]. Notably, higher PGPR application rates triggered more pronounced structural shifts, particularly within bacterial communities, with earlier reports showing that PGPR inoculation can lead to dominant bacterial guild replacement and rhizosphere restructuring in cereal crops [36].

Regarding community stability, bacterial community stability increased significantly with higher PGPR application rates (Figure 5a), suggesting enhanced bacterial robustness and resilience against disturbance. This pattern is consistent with the insurance hypothesis, which posits that increased microbial diversity enhances community resilience to environmental perturbations [37]. In contrast, fungal community stability peaked under B1 treatment but declined slightly under B2 (Figure 5b), implying that excessive PGPR application may impose competitive or stress pressures on fungal communities [38].

### 3.4. Effects of PGPR on Comprehensive Oat Growth Through Coordinated Regulation of Soil and Microbial Communities

A comprehensive growth index was employed to evaluate the effects of different PGPR application rates on overall oat growth performance and to further elucidate the respective contributions of soil physicochemical properties and rhizosphere microbial communities.

Both B1 and B2 treatments significantly improved the comprehensive growth index relative to the control (*p* < 0.001; Figure 6a), with no significant difference observed between the two treatments (*p* > 0.05; Figure 6a). Although root exudates were not directly measured in this study, the observed growth promotion is consistent with earlier reports suggesting that rhizosphere bacteria may enhance plant performance partly by modulating root exudate composition [39]. These results suggest that an appropriate PGPR application rate is sufficient to achieve optimal growth promotion, whereas further increases in application rate may not result in additional benefits. Similar patterns of diminishing returns with excessive microbial input have been reported in other systems [40]. The observed initial increase followed by a decline in the comprehensive growth index may reflect microecological disturbances in the rhizosphere caused by excessive PGPR application or resource reallocations between nutrient uptake and vegetative growth [41,42]. Further studies are warranted to elucidate the regulatory mechanisms underlying these interactions, particularly those involving hormonal crosstalk and plant–microbiome co-adaptation [43]. The mixed-effects model revealed that soil and microbial factors were the key determinants of plant growth performance. Together, they explained 81% of the variance in the comprehensive growth index (_Mar_R^2^ = 0.81; Figure 6b), with soil factors contributing 53.37% and microbial factors 46.63%. This finding suggests that the growth-promoting effects of PGPR depend not only on its intrinsic biological activity but also on the synergistic regulation of soil conditions and microbial community dynamics. Specifically, fungal community stability, fungal β-diversity, and soil organic matter (OM) were identified as key positive drivers (*p* < 0.05; Figure 6b), which is consistent with studies showing that PGPR and bio-organic amendments significantly enhance fungal β-diversity and organic matter content, thus improving soil ecosystem functions and nutrient availability [44,45], whereas nitrate nitrogen (NO_3_^−^-N) exerted a significant negative effect (*p* < 0.01; Figure 6b), suggesting that excessive nitrate may disrupt microbial balance or suppress beneficial fungi and nitrogen-cycling organisms, as also observed in rhizosphere environments under high N-input regimes [46,47]. These results highlight the pivotal role of rhizosphere fungi in enhancing system stability and facilitating resource cycling. Unlike short-lived and free-living bacteria, fungi exert stable, structural influences on plant community ecology across broader spatial and temporal scales [48]. Furthermore, the positive role of soil organic matter, a core indicator of soil quality, underscores the importance of PGPR-mediated improvements in soil physicochemical properties [32].

Structural equation modeling (SEM) revealed that PGPR directly enhanced comprehensive oat growth and also exerted indirect effects through both soil and microbial pathways, with the soil-mediated pathway showing a slightly stronger influence (β = 0.437 * vs. β = 0.424 **; Figure 7a). This finding aligns with prior work indicating that rhizosphere manipulation by PGPR enhances crop performance both via direct stimulation and by improving soil biochemical characteristics and microbial network stability under saline conditions [21,49]. Quantitative analysis of pathway contributions showed that PGPR had the greatest total effect on the comprehensive growth index (0.74), followed by soil properties (0.44) and microbial communities (0.42) (Figure 7b). Similar SEM-based frameworks have demonstrated that rhizosphere management, particularly through PGPR and AMF, can shift community assembly and metabolite availability in favor of plant growth under stress [50,51]. Overall, these findings demonstrate that PGPR enhances oat growth not only through direct promotion but also by establishing a systemic enhancement mechanism via the coordinated optimization of the rhizosphere soil and microbial environment under saline soil conditions in the Qaidam Basin.

## 4. Materials and Methods

### 4.1. Experimental Site Description and PGPR Preparation

The field experiment was conducted at the experimental site of Bensheng Forage Company, located at Gahai Town, Delingha City, Haixi Mongolian and Tibetan Autonomous Prefecture, Qinghai Province, China (37°15′12.84027″ N, 97°22′39.75037″ E), at an elevation of 2843.30 m (Supplementary Figure S1). The site covers an area of 6.67 hm2 and is characterized by cold temperatures, low oxygen levels, dry air, limited precipitation, and strong winds. The site is equipped with a mobile sprinkler irrigation system to ensure adequate irrigation conditions. The preceding crop was ‘Qingtian No. 1’ oat (Avena sativa).

Isolation and characterization of PGPR strains: Bacterial strains used in this study were isolated from soil samples collected from oat cultivation fields and saline–alkali soils located in Haibei Prefecture and Delingha City (Qinghai Province), as well as Zhangye City and Gannan Prefecture (Gansu Province), China. Isolation was performed by serial dilution method, where diluted soil suspensions were plated onto Luria-Bertani (LB) agar medium and incubated at 28 °C for 24–48 h until discrete colonies appeared. Pure cultures were isolated by repeated streaking on fresh LB agar, verified morphologically, and stored at −80 °C in 20% glycerol.

Screening for plant growth-promoting traits and salt tolerance: Preliminary screening involved evaluating the isolates’ plant growth-promoting capabilities and salt tolerance. For plant growth assays, oat seeds provided by the Qinghai Grassland Improvement Experimental Station were surface-sterilized, immersed in bacterial suspensions (10^8^ CFU mL^−1^) for 30 min, and then transferred onto sterile germination trays containing moist sterile filter paper. Seedlings were cultured in a growth chamber at 28 °C under a 12 h photoperiod, with daily additions of sterile water to maintain adequate moisture. On day 7, seedlings were thinned to eight seedlings per tray and supplemented with 1 mL of half-strength Murashige and Skoog nutrient medium to sustain seedling growth. After a total incubation of 15 days, plant height, root length, and fresh biomass of shoots and roots were measured to evaluate growth-promoting efficiency.

Salt tolerance was assessed by inoculating 100 µL aliquots of overnight bacterial cultures (approximately 10^8^ CFU mL^−1^) into 10 mL LB broth containing graded concentrations of NaCl (1%, 3%, 5%, and 7%). Cultures were incubated at 30 °C on a rotary shaker (200 rpm), and bacterial growth was monitored at optical density (OD_600 nm) intervals of 2 h over a period of 24 h. Based on superior performance in both promoting plant growth and exhibiting robust salt tolerance, two isolates designated GN-1 and MQ-5 were selected for further characterization.

Determination of specific plant growth-promoting traits: The selected strains GN-1 and MQ-5 were further evaluated for specific plant growth-promoting characteristics using standard procedures, including biological nitrogen fixation capability (acetylene reduction assay), phosphate solubilization (using Pikovskaya’s agar medium), potassium solubilization (on Aleksandrov agar medium), indole-3-acetic acid (IAA) production (quantified by the Salkowski method), and siderophore production (assessed using chrome azurol S assay).

Molecular identification of selected strains: Genomic DNA from GN-1 and MQ-5 was extracted using the DNeasy UltraClean Microbial DNA Isolation Kit (Qiagen, Hilden, Germany). The 16S rRNA gene was amplified via PCR using universal primers 27F (5′-AGAGTTTGATCMTGGCTCAG-3′) and 1492R (5′-GGTTACCTTGTTACGACTT-3′). PCR products were purified and sequenced on an ABI 3730xl DNA Analyzer (Applied Biosystems, Foster City, CA, USA). The resulting sequences were compared to known bacterial sequences in the GenBank database using BLASTn (BLAST+ suite v2.14.0; National Center for Biotechnology Information, Bethesda, MD, USA). Strain GN-1 was identified as *Bacillus mycoides*, while MQ-5 was identified as *Bacillus* sp.

Preparation of PGPR inoculum formulation: For inoculum preparation, selected strains GN-1 (*Bacillus mycoides*) and MQ-5 (*Bacillus* sp.) were individually streaked onto LB agar plates (composition per liter: tryptone 10 g, yeast extract 5 g, NaCl 10 g, agar 20 g; pH 7.0 ± 0.2) and incubated at 37 °C for 24 h to obtain actively growing colonies. Activated single colonies were subsequently inoculated into LB broth medium (composition per liter: tryptone 10 g, yeast extract 5 g, NaCl 10 g; pH 7.0 ± 0.2) and incubated at 37 °C on a rotary shaker at 150–200 rpm for 24 h until reaching stationary phase with sufficient cell density (~10^9^ CFU mL^−1^). Bacterial suspensions obtained were appropriately diluted or concentrated according to subsequent experimental needs and uniformly mixed as required for inoculum application.

The PGPR inoculant used in this study consisted principally of two spore-forming strains, *Bacillus* sp. and *Bacillus mycoides*. The key physicochemical properties of this biofertilizer are summarized in Supplementary Table S1. Prior to sowing, the PGPR inoculants were activated. Field-collected soil (500 g) was adjusted to a water content of 65% to 70% to simulate rhizosphere conditions. PGPR inoculants were thoroughly mixed into the soil at three application rates: control (CK, 0 kg·hm^−2^), B1 (75 kg·hm^−2^, equivalent to 7.5 g·m^−2^), and B2 (150 kg·hm^−2^, equivalent to 15 kg·hm^−2^). The mixtures were pre-incubated at 25 °C for 7 days to promote initial colonization and activation before field application. At sowing, the activated PGPR inoculants were evenly distributed into furrows with a depth of 3–4 cm and a row spacing of 30 cm, followed by the sowing of oat seeds. The furrows were subsequently covered with soil and manually compacted.

### 4.2. Experimental Design and Field Management

Oat cultivar ‘Qingtian No. 1’ (germination rate > 90%, seed purity ≥ 90%, provided by Qinghai Kai rui Ecological Technology Co., Ltd. (Xining, Qinghai, China)) was sown manually by furrow drilling at a depth of 3–4 cm on 9 May 2023. The sowing rate was 300 kg·hm^−2^. Prior to sowing, the soil was mechanically tilled, and basal fertilizers consisting of organic fertilizer (300 kg·hm^−2^) and compound chemical fertilizer (375 kg·hm^−2^; N:P_2_O_5_:K_2_O = 25:12:5) were uniformly applied. Weed control was conducted twice during the seedling stage, and mobile sprinklers provided supplemental irrigation according to soil moisture conditions.

A randomized block design with three treatments was adopted: CK (no inoculant applied), B1 (75 kg·hm^−2^ inoculant), and B2 (150 kg·hm^−2^ inoculant). Each treatment comprised three replicate plots (5 m × 3 m each, total area 15 m^2^ per plot), spaced 50 cm apart, resulting in a total of nine plots. According to Chinese agricultural industry standards (NY/T 798), an inoculant application rate of 75 kg·hm^−2^ is recommended to effectively meet crop microecological needs. Given the low inherent soil fertility, specific sandy soil texture, saline soil conditions [52], and alpine arid environment at the experimental site [53], the inoculant application rate was further increased to 150 kg·hm^−2^ to maximize potential plant-growth-promoting effects under these challenging conditions.

### 4.3. Plant Growth Assessment

Plant height was measured at maturity using a ruler graduated to 0.01 cm from the stem base to the tip of the uppermost leaf or panicle. Ten randomly selected plants per plot were recorded. Aboveground biomass was determined by harvesting a 50 cm row segment per plot at ground level, recording fresh mass immediately in the field, and then oven-drying the samples at 105 °C for 30 min, followed by 65 °C until a constant weight was achieved for dry mass determination. Leaf chlorophyll content was quantified on the flag leaf with a portable chlorophyll meter (SPAD-502 Plus; Konica Minolta Co., Ltd., Osaka, Japan). Three evenly spaced points along the mid-section of each leaf were measured in triplicate, and the mean value was recorded as the SPAD index. Because the SPAD index is strongly and positively correlated with the concentration of chlorophyll a + b per unit leaf area, it serves as a rapid, nondestructive proxy for evaluating crop photosynthetic capacity. For root traits, ten uniform plants per plot were excavated using the trench method, gently rinsed, blotted dry, scanned with a flatbed root scanner (Perfection V700 Photo; Seiko Epson Corporation, Suwa, Nagano, Japan), and analyzed using WinRHIZO Pro v2019c (Regent Instruments Inc., Québec City, QC, Canada) to obtain total root length, root surface area, root volume, and root tip number.

### 4.4. Soil and Rhizosphere Sampling

At maturity, vegetation status was inspected, and six cores (38 mm diameter, 0–20 cm) were taken from uniform zones of each plot. Loosely adhering soil was removed, and the 0–5 mm soil still attached to roots was collected as rhizosphere soil, sealed in sterile bags, and transported at −20 °C. Aliquots were air-dried, sieved (0.25 mm), and analyzed for physicochemical properties: pH (soil/water = 1:2.5) with a PHS-3C pH-meter [54]; organic matter (OM) by external-heat dichromate oxidation [55]; total N (TN) by semi-micro Kjeldahl [56]; total P (TP) by molybdo-antimony colorimetry [57]; nitrate-N (NO_3_^−^-N) by UV spectrophotometry [58]; and ammonium-N (NH_4_^+^-N) by Nessler’s reagent colorimetry [59].

### 4.5. Rhizosphere DNA Sample Preparation

Root systems bearing firmly attached soil were placed in sterile 50 mL tubes containing 30 mL autoclaved PBS (0.1% Tween-80, pH 7.0). Samples were sonicated for 10 min, the suspension was transferred to fresh sterile tubes, and the procedure was repeated three times. Combined suspensions were centrifuged at 6000 rpm for 5 min; the pellets were freeze-dried at −40 °C for ≥12 h and stored at −80 °C until DNA extraction [60].

### 4.6. Rhizosphere Microbial DNA Extraction, Amplicon Library Construction, and Bioinformatic Analysis

Total genomic DNA was isolated from 0.5 g of rhizosphere soil using the Mo Bio PowerSoil kit (QIAGEN Inc., Germantown, MD, USA) following the manufacturer’s protocol. The bacterial 16S rRNA V4 region was amplified with primers 515MF/907R, while the fungal ITS2 region was amplified with primers 5.8F/4R. Amplicons were purified, quantified with a Qubit 3.0 fluorometer (Thermo Fisher Scientific Inc., Waltham, MA, USA; Qubit Software v3.2.0), and pooled equimolarly. Dual-indexed paired-end (PE 250) libraries were prepared with the NEXTFLEX Rapid DNA-Seq Kit v14-06-21 (PerkinElmer Inc., Austin, TX, USA) and sequenced on an Illumina MiSeq platform (Shanghai LingEn Biotech Co., Shanghai, China). Raw reads were quality-trimmed (sliding-window 10 bp; Q < 20), reads < 50 bp were discarded, and paired reads were merged with a minimum 10 bp overlap allowing ≤20% mismatches. Chimera filtering yielded high-quality sequences that were de-replicated and denoised with UNOISE to generate amplicon sequence variants (ASVs) at 97% similarity. Taxonomic assignment of representative ASVs was performed with the RDP classifier (confidence ≥ 0.7) against the SILVA 138.2 database. The resulting ASV table provided the basis for subsequent diversity and community-composition analyses.

### 4.7. Statistical Analyses

To assess differences in plant growth traits, rhizosphere soil physicochemical properties, and rhizosphere microbial diversity and stability among treatments, data were first tested for normality and homogeneity of variance. Pairwise comparisons were then performed using independent two-sample *t*-tests, and Bonferroni correction was applied to adjust the final *p*-values. Alpha diversity of rhizosphere microbial communities was assessed using the richness index, Shannon index, Pielou index, and phylogenetic diversity (PD), with PD calculated via the picante package in R [61]. Community structure differences were visualized using non-metric multidimensional scaling (NMDS) based on Bray–Curtis dissimilarity, calculated via the vegan package [62]. PERMANOVA was used to test group-level differences, with *p*-values adjusted using Bonferroni correction [63]. Microbial community stability was assessed with the average variation degree (AVD) method [64]. After rarefying all samples to an equal sequencing depth, we quantified, for every OTU in each replicate, the absolute deviation of its abundance from the treatment mean and normalized this deviation by the OTU-specific standard deviation. These normalized deviations were then averaged across all OTUs and replicates to obtain a single AVD value per treatment, where lower AVD indicates greater stability. For ease of interpretation, we used the reciprocal of AVD (RAVD = 1/AVD) as the final stability index, so higher RAVD values represent more stable microbial communities.

For an integrated assessment of oat performance, six traits—plant height, aboveground biomass, belowground biomass, total root length, root surface area, and root volume—were standardized as Z-scores. A comprehensive growth index (CG) was calculated by averaging the Z-scores [65], following the multifunctionality index approach used in ecosystem studies [66,67].

To identify key drivers of the CG index, variance inflation factors (VIFs) were calculated to exclude collinear predictors (VIF > 10). Subsequently, all-subset regression with AIC-based model selection was conducted using the MuMIn package, incorporating the plot as a random effect [68]. The relative contributions of predictors to the variance explained (marginal R^2^) were estimated via hierarchical partitioning using the glmm.hp package [69]. Data visualizations were performed using the ggplot2 package.

Finally, based on the optimal model selected by all-subsets regression, a piecewise structural equation model (SEM) was constructed using the piecewiseSEM package [70] to elucidate key causal pathways linking rhizosphere soil, microbial communities, and oat growth under alpine conditions. Model fit was considered acceptable when *p* > 0.05. Lower Akaike Information Criterion (AIC) and Fisher’s C statistics indicated better model performance and explanatory power [71].

Statistical analyses and data visualization were performed using R (version 4.4.1).

## 5. Conclusions

We systematically evaluated the effects of different application rates (B1 = 75 kg·hm^−2^ and B2 = 150 kg·hm^−2^) of plant growth-promoting rhizobacteria (PGPR) on oat growth, soil physicochemical properties, and rhizosphere microbial communities, thereby elucidating the roles of PGPR in regulating plant–soil–microbe interactions. The results demonstrated that PGPR application significantly enhanced oat growth, increased productivity, raised the comprehensive growth index, augmented soil organic matter content, and reduced soil pH, exhibiting clear dose-dependent effects. Specifically, B1 treatment favored aboveground biomass accumulation, fungal community stability, soil organic matter enrichment, and soil pH reduction, whereas B2 treatment more strongly promoted root system development, bacterial community stability, soil organic matter accumulation, and soil pH regulation. Further analysis identified soil organic matter content, fungal β-diversity, and the community stability of both bacteria and fungi as key positive contributors to the comprehensive growth performance of oats. Collectively, these findings provide a theoretical basis for the scientific application and dosage optimization of PGPR in alpine forage production and lay a foundation for promoting the efficient use of microbial fertilizers in agriculture under saline soil conditions in the Qaidam Basin.

## Data Availability

All sequencing data have been deposited in the National Microbiology Data Center (NMDC) under accession number NMDC10019855 (https://nmdc.cn/resource/genomics/project/detail/NMDC10019855).

## Supplementary Materials

The following supporting information can be downloaded at https://www.mdpi.com/article/10.3390/plants14131926/s1, Figure S1: Experimental site distribution map; Table S1: Physicochemical characteristics of PGPR fertilizer.
