# Supplementary material for: Rhizosphere Growth-Promoting Bacteria Enhance Oat Growth by Improving Microbial Stability and Soil Organic Matter in the Saline Soil of the Qaidam Basin
Source: Plants (Basel). 2025 Jun 23;14(13):1926. doi: 10.3390/plants14131926 (PMC12252047; doi:10.3390/plants14131926)
Supplement: Supplementary file 1 [file plants-14-01926-s001.zip › plants-3666421-supplementary.pdf]

Supplementary materials for

Rhizosphere growth-promoting bacteria enhance oat growth by improving microbial stability and soil organic matter in the saline soil of the Qaidam Basin

Xin Jin †, Xinyue Liu †, Jie Wang , Jianping Chang , Caixia Li , Guangxin Lu *

College of Agriculture and Animal Husbandry, Qinghai University, Xining 810016, China; 18894310895@163.com (X.J.); 17856838703@163.com (X.L.); wangjie422022@163.com (J.W.); c223663@126.com (J.C.); lcxia1314@126.com (C.L.)
* Correspondence: lugx74@163.com; Tel.: +86-13897216290
† These authors contributed equally to this work.

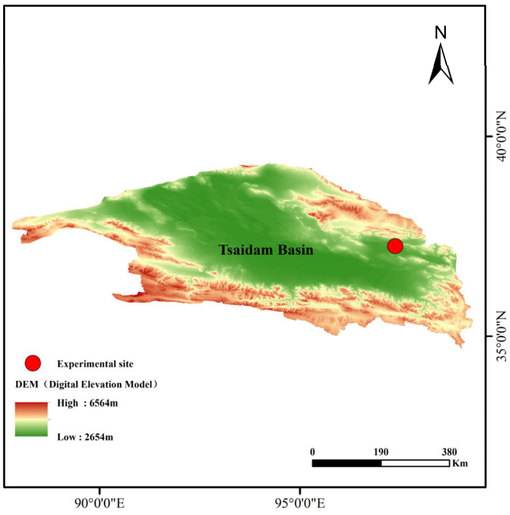

Figure S1. Experimental site distribution map.

Table S1. Physicochemical Characteristics of PGPR Fertilizer

Test items	Test result	Unit
Total number of effective viable bacteria(Bacillus count)	7.80	10^8CFU • g^{-1}
Total number of effective viable bacteria	7.80	10^8CFU • g^{-1}
Fecal coliforms	<0.3	MPN • g^{-1}
Moisture	33.2	%
Organic matter (on a dry basis)	89.8	%
pH	8.2	/
